# Probing Protein Glycation by Chromatography and Mass Spectrometry: Analysis of Glycation Adducts

**DOI:** 10.3390/ijms18122557

**Published:** 2017-11-28

**Authors:** Alena Soboleva, Maria Vikhnina, Tatiana Grishina, Andrej Frolov

**Affiliations:** 1 Department of Biochemistry, St. Petersburg State University, 199034 Saint Petersburg, Russia; st021585@student.spbu.ru (A.S.); vikhnina@gmail.com (M.V.); tgrishina@mail.ru (T.G.); 2 Department of Bioorganic Chemistry, Leibniz Institute of Plant Biochemistry, 06120 Halle (Saale), Germany

**Keywords:** advanced glycation end products (AGEs), amino acid analysis, exhaustive hydrolysis, glycation, glycation adducts, glyoxalase, LC-MS/MS, stable isotope dilution, standard addition

## Abstract

Glycation is a non-enzymatic post-translational modification of proteins, formed by the reaction of reducing sugars and α-dicarbonyl products of their degradation with amino and guanidino groups of proteins. Resulted early glycation products are readily involved in further transformation, yielding a heterogeneous group of advanced glycation end products (AGEs). Their formation is associated with ageing, metabolic diseases, and thermal processing of foods. Therefore, individual glycation adducts are often considered as the markers of related pathologies and food quality. In this context, their quantification in biological and food matrices is required for diagnostics and establishment of food preparation technologies. For this, exhaustive protein hydrolysis with subsequent amino acid analysis is the strategy of choice. Thereby, multi-step enzymatic digestion procedures ensure good recoveries for the most of AGEs, whereas tandem mass spectrometry (MS/MS) in the multiple reaction monitoring (MRM) mode with stable isotope dilution or standard addition represents “a gold standard” for their quantification. Although the spectrum of quantitatively assessed AGE structures is continuously increases, application of untargeted profiling techniques for identification of new products is desired, especially for in vivo characterization of anti-glycative systems. Thereby, due to a high glycative potential of plant metabolites, more attention needs to be paid on plant-derived AGEs.

## 1. Introduction

**Figure 1:**
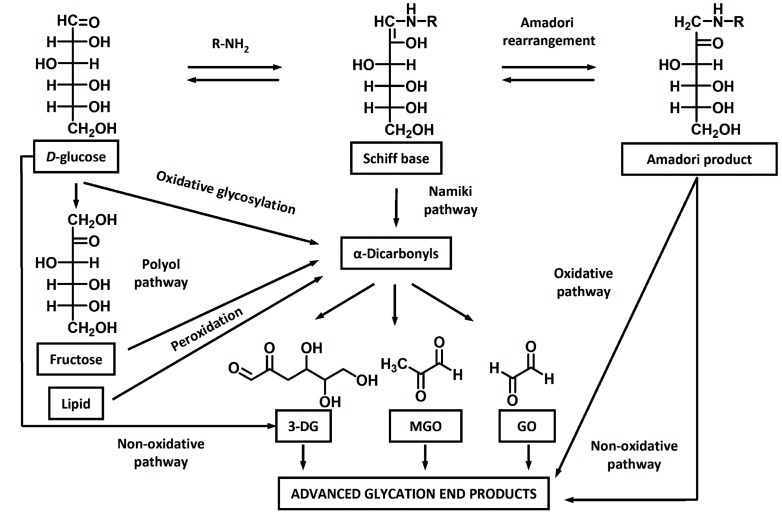
The major pathways of AGE formation: oxidative glycosylation [12], Namiki pathway [13], enolization [14], oxidative [15] and non-oxidative [16] degradation of early glycation products, polyol [17], and lipid peroxidation [18]. 3-DG, 3-deoxyglucasone; GO, glyoxal; MGO, methylglyoxal.

**Figure 2:**
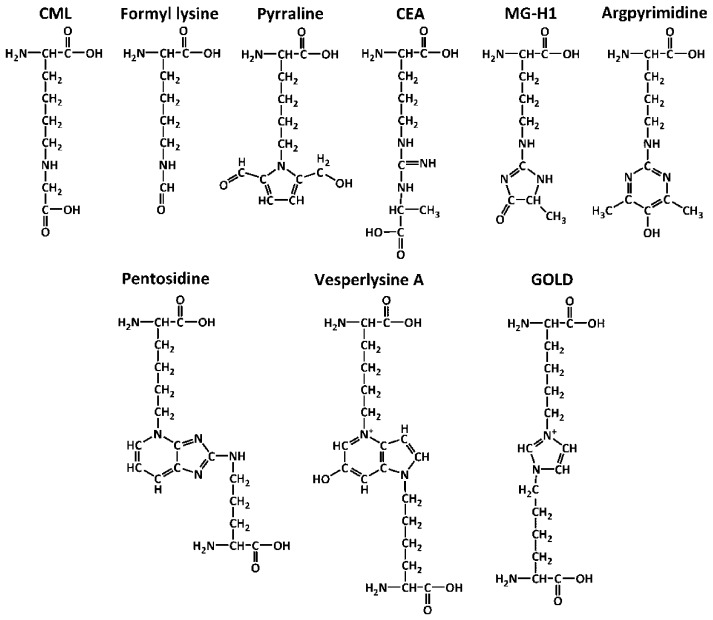
Advanced glycation end products (AGEs) originating from lysine (*N^ε^*-(carboxymethyl)lysine, CML; *N^ε^*-(formyl)lysine; pyrraline), arginine (*N^δ^*-(carboxyethyl)arginine, CEA; *N^δ^*-(5-methyl-4-oxo-5-hydroimidazo-linone-2-yl)ornithine, MG-H1; argpyrimidine) and of cross-link nature (pentosidine; vesperlysine A; glyoxal-derived lysine dimer, GOLD).

Glycation is a non-enzymatic post-translational modification of proteins with reducing sugars and α-dicarbonyl products of their degradation [1]. In the first step (early glycation), reducing sugars, aldoses and ketoses, react with amino groups yielding aldimines and ketoimines (Schiff bases), which are readily involved in Amadori and Heyns rearrangements, yielding 1-amino-deoxyketosyl and 2-amino-deoxyaldos-2-yl adducts [2,3]. These early glycation products are involved in further oxidative (glycoxidation) and non-oxidative degradation (Figure 1), yielding a heterogeneous group of advanced glycation end-products (AGEs, Figure 2) [4,5]. AGEs can be also formed by the “oxidative glycosylation” pathway [6], via interaction of lysyl and arginyl residues with α-dicarbonyls, like glyoxal (GO), methylglyoxal (MGO), and 3-deoxyglucasone (3-DG) [7]—the intermediates of monosaccharide autoxidation [8], lipid peroxidation [9], polyol pathway [10], and non-enzymatic conversion of triosophosphates [11].

Generally, glycation can occur internally, i.e., in animal (human) [16,19,20], plant [21,22], and bacterial [23] organisms, or externally—during thermal processing of foods [24]. Accompanying accumulation of AGEs in human body results in cross-linking of long-living proteins, like crystallines and collagens [25]. Moreover, interaction of AGEs with multi-ligand immunoglobulin-like receptors (e.g., RAGEs—receptors for advanced glycation end products) triggers nuclear translocation of the transcription factor NF-κB and induction of inflammation-specific genes [26]. In turn, it results in development of sub-clinical systemic inflammation [27], which impacts in atherosclerosis [28], ageing [29], neurodegenerative disorders, like Alzheimer and Parkinson diseases [30,31], diabetes mellitus (DM) and its complications [32,33,34,35,36,37,38,39,40].

Among lysine-derived AGEs, *N^ε^*-(carboxymethyl)lysine (CML) and *N^ε^*-(carboxyethyl)lysine (CEL), formed via both glycoxidative and autoxidative pathways, are the most well-studied representatives [41,42], whereas interaction of lysyl residues with 3-DG, 3-deoxypentosone (3-DP), and glyceraldehyde yield pyrraline, formyline, and glyceraldehyde-derived pyridinium (GLAP), respectively (Figure 2) [43,44,45]. Recently, Glomb and co-workers characterized a group of amide AGEs, represented by *N^ε^*-glycoloyl-, -formyl-, -acetyl-, -glycerinyl(lysine), *N^ε^*-[2-[(5-amino-5-carboxypentyl)amino]-2-oxoethyl]lysine (GOLA) [14,46], and confirmed its clinical relevance [19]. In human tissues, arginine-related AGEs are dominated with GO-derived (1-(4-amino-4-carboxybutyl)-2-imino-5-oxo-imidazolidine, Glarg) [47], and three MGO-derived hydroimidazolones-*N^δ^*-(5-methyl-4-oxo-5-hydroimidazo-linone-2-yl)-l-ornithine (MG-H1, the major adduct) [48], 2-amino-5-(2-amino-5-hydro-5-methyl-4-imidazolon-1-yl)pentanoic acid (MG-H2) and 2-amino-5-(2-amino-4-hydro-4-methyl-5-imidazolon-1-yl)pentanoic acid (MG-H3) [49]. Under alkaline conditions, Glarg and MG-H3 can be hydrolyzed to yield *N^δ^*-carboxymethyl- (CMA) and *N^δ^*-(carboxyethyl)arginine (CEA), respectively [50,51]. Modification with two MGO molecules yields *N^δ^*-(5-hydroxy-4,6-dimethylpyrimidine-2-yl)-l-ornithine (argpyrimidine) [52] and *N^δ^*-(4-carboxy-4,6-dimethyl-5,6-dihydroxy-1,4,5,6-tetrahydropyrimidine-2-yl)-l-ornithine (tetra-hydropyrimidine, THP) [53]. Cross-linking is an essential feature of advanced glycation (Figure 2). In this context, pentosidine was identified at the late 1980s as the first cross-link AGE [54]. Later, crossline and vesperlysines A, B, and C were reported as modifications of lens proteins under hyperglycemic conditions [55,56,57]. Reaction of α-dicarbonyls, i.e., GO, MGO, and 3-DG with two lysine residues result in formation of imidazolium cross-links, i.e., glyoxal-, methylglyoxal-, and 3-deoxyglucasone-derived lysine dimers (GOLD, MOLD, and DOLD, respectively) [58,59].

As glycation products are recognized as the markers of food quality [24], ageing [37] and metabolic diseases [60,61,62], numerous analytical approaches were established to address their contents in corresponding matrices. Thus, Amadori-modified proteins (e.g., glycated hemoglobin HbA_1c_) can be effectively separated from unglycated counterparts by cation exchange chromatography (CXC) [63], and selectively enriched by boronic acid affinity chromatography (BAC) [64,65], or its combination with immunochemical methods, for example, enzyme-linked boronate-immunoassay (ELBIA) [66,67]. In the easiest way, AGEs can be quantified spectrophotometrically by a characteristic increase in absorbance (300–400 nm) [68,69] or by fluorescence at the excitation and emission wavelengths of 370 and 440 nm, respectively [70,71]. However, both these techniques lack specificity, and do not provide information about individual AGE classes, that dramatically reduces their diagnostic potential. Alternatively, this kind of information can be delivered by immunoassays. However, these techniques suffer from a high degree of non-specific binding and typically do not allow simultaneous quantification of several AGEs [72,73].

In this context, implementation of mass spectrometry (MS) in analysis of protein glycation products dramatically increases its sensitivity, selectivity, precision, and robustness [74,75]. Indeed, it provides an effective tool for structural characterization and quantification of individual early and advanced glycation products on the levels of individual glycated amino acids [76], peptides [77,78], and proteins [79,80]. Currently, these techniques are being effectively introduced in food quality control [81,82] and medical diagnostics [83,84,85]. Here we provide a comprehensive review of existing chromatographic and mass spectrometric techniques used for characterization of protein glycation adducts, i.e., analytical approaches relying on the methods of amino acid analysis. Thereby we consider individual protein-derived and free amino acids as the targets of MS analysis and discuss them in the context of the actual trends in Maillard research.

## 2. Methods of Amino Acid Analysis in Glycation Research

Generally, analysis at the level of individual amino acids (either free, or obtained by exhaustive hydrolysis of proteins) is the most straightforward and direct way to characterize the structures and quantities of glycation products, occurring in artificial and natural systems [86]. Therefore, the employed analytical strategies most commonly rely on amino acid analysis protocols, and provide a direct access to absolute quantities of individual glycation adduct classes (as well as unmodified amino acids) in biological samples of various origin and complexity [65]. Thereby, different experimental setups allow identification of individual modifications and quantification of glycation rates in proteins, as well as the products of their in vivo hydrolysis (so-called “glycation free adducts”) [74]. This workflow proved to be well-compatible with physiological experiments, performed at the molecular [87], cellular [88] or organism [76] levels, and applicable to in vitro model experiments and receptor affinity studies [89]. Whereas the early works addressed mostly simple model glycation systems (with consideration of only Amadori compound and CML as the major products), later studies employed multistep enzymatic hydrolysis protocols and covered a wide panel of glycation and oxidation products [74]. The main landmarks, indicating development of Maillard reaction analytics, based on amino acid analysis, are summarized in Table 1.

### 2.1. GC-MS Analysis of Free Glycated Amino Acids

In the early model glycation studies, gas chromatography (GC) with flame ionization detection (FID) was used for analysis of acetylated CML derivatives [90] (Table 1). Alternatively, detection could be performed by electron (impact) ionization mass spectrometry (EI-MS), which provided reliable confirmation of the compound structures by characteristic fragmentation patterns [41]. From another hand, CML could be successfully analyzed in the form of trifluoroacetyl methyl esters [91] or as isobutyl alcohol-pentafluoropropionic anhydride derivatives [92]. Additional information about the structures of AGE derivatives could be obtained by the combination of chemical ionization and tandem mass spectrometry (MS/MS) [92], whereas selected ion monitoring (SIM) provided higher sensitivity and specificity in the analysis of serum free glycated adducts [93].

### 2.2. Exhaustive Degradation of Proteins to Obtain Amino Acid Glycation Adducts

Obviously, for analysis of glycation adducts in proteins, the polypeptide chain needs to be degraded to give access to individual monomers. In the simplest way, it can be achieved by pyrolysis GC-MS (Py-GC-MS). Thus, based on the experiments with polylysine [94], Lapolla and co-workers proposed this technique as a potential tool for DM diagnostics, and demonstrated clear differences of pyrolysis profiles obtained for glycated albumin from those, acquired with untreated protein [95] (Table 1). However, this approach has at least two essential disadvantages: from one hand, high temperatures, applied for degradation of the polypeptide chain, result in degradation of glycated amino acid side chains (that might lead to the loss of structural information), from another, the pattern of resulted pyrolysis cleavage products is difficult to interpret.

**Figure 3:**
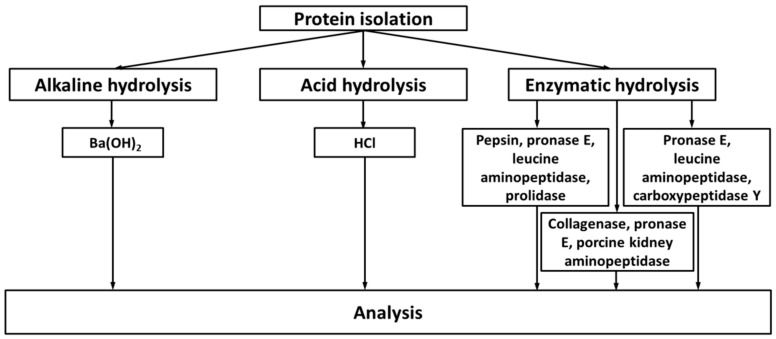
The overview of protein hydrolysis workflows (alkaline [99], acid [19,115] and enzymatic [19,115,116]), compatible with subsequent chromatography-based analysis. Concentrations: Ba(OH)_2_, 1.7 mol/L [99]; HCl, 6 N [19,115]; pronase E, two additions of 0.3 unit [19], 400 PU [115], 20 μg [116]; leucine aminopeptidase, 1unit [19], 0.4 unit [115]; carboxypeptidase Y, 0.95 unit [19]; pepsin, 1 FIP-U [115]; prolidase, 1 unit [115]; collagenase, 0.04 mg/mL [116].

In this context, exhaustive degradation of proteins, yielding free amino acids, seems to be an advantageous technique (Figure 3). In the easiest and the most straightforward way, it can be achieved by acid hydrolysis in presence of 6 N HCl at 100–110 °C during 18–24 h [98,102] (Table 1). The ease of the experimental setup, high reliability, reproducibility, and quantitative character of hydrolysis are the obvious advantages of this technique. Because of this, the method is often applied to quantification of CML in foods or biological matrices. For example, the contents of CML and pyrraline were addressed as the indicators of advanced glycation during heat treatment of carrots [104]. Analogously, HPLC-ESI-MS was applied to quantification of CML in various dairy products [105,106,107]. However, acid hydrolysis has some intrinsic limitations, which need to be kept in mind when applying it to analysis of glycation adducts. First, incubation at high temperatures results in a rapid glycoxidative degradation of Amadori moieties, already present in the protein sequence before incubation [108], that ultimately results in overestimation (up to 12 times) of CML [100]. To make an appropriate correction for the generation of CML during sample preparation, hydrolysis is additionally performed after a pre-incubation of the protein with NaBH_4_—a strong reducing reagent, readily converting Amadori and Heyns compounds in corresponding alcohols [107]. From the other hand, incubation at high temperatures and low pH results in degradation of arginine-derived hydroimidazolones (such as Glarg, MG-H, and 3DG-H), their precursors (corresponding hydroxyimidazolidinones) and products (CMA, CEA, Argpyr and THP). At least, for hydroimidazolones this degradation can reach 90% under the acid hydrolysis conditions [49]. Finally, under these conditions, Amadori compounds degrade to form *N^ε^*-(2-furoyl-methyl)-l-lysine (furosine) [101] and hydroxymethylfurfural [109], the products known to accompany thermal degradation of *N^ε^*-(fructosyl)lysine (Amadori compounds) in foods since more than fifty years [110,111,112]. Although the formation of furosine is temperature-dependent, it is successfully used in food chemistry for estimation of “blocked” (i.e., glycated) lysine residues in proteins [113]. In the most reliable way it can be done by LC-MS or MS/MS using a standard isotope dilution approach [96,114]. However, for biological applications, enzymatic hydrolysis is the method of choice [74]. Thus, in medical and food research, acid hydrolysis is currently presumably used for normalization of analysis by determination of unmodified amino acids, but not for quantitative assessment of glycation adducts [74].

In some cases, the limitations of acid hydrolysis can be overcome by employing protein degradation under alkaline conditions in presence of barium hydroxide, as it was done by Portero-Otin et al. for quantification of pyrraline in proteins, glycated in vitro (bovine serum albumin (BSA) and collagen) and in plasma [99] (Table 1). Application of this method, however, might result in a strong underestimation of Glarg and MG-H3 due to their high susceptibility to alkali hydrolysis, accompanied with conversion of these imidazolones in CMA and CEA, respectively [46,117]. Thus, enzyme-based techniques of protein degradation are the methods of choice for analysis of protein-bound glycation adducts [74]. Indeed, currently, exhaustive enzymatic hydrolysis allows reliable quantification of dozens of individual glycation adducts formed by free amino acids, proteins, and nucleic acids [76].

Typically, the hydrolysis procedure relies on a sequential treatment of a dissolved protein sample with individual proteinases, or their combinations. For example, Glomb and co-workers proposed several robust and reliable protocols, relying on sequential treatment with proteinase K, pronase E, amino- and carboxypeptidase (24-h incubations) [46] (Table 1, Figure 3). According to the protocol established in Henle’s lab, this procedure can be prefaced by an incubation with pepsin, whereas the last reaction can be complemented with prolidase (Figure 3) [115,118]. For specific proteins, treatment with additional appropriate protease(s) can be introduced. For example, Iijima and co-workers replaced pepsin with collagenase when analyzing in vitro collagen glycation mixtures (Figure 3) [116]. For urine samples, an acylase treatment, to assess *N*-acetyl amino acid conjugates, can be additionally applied [65]. Later on, some further extensions of this protocol were done by Thornalley and co-workers. For example, pepsin can be omitted in analysis of apolipoprotein B100, whereas digestion of hemoglobin can be performed under carbon monoxide to prevent artefactual heme-catalyzed glycoxidation [74].

When setting up enzymatic hydrolysis, it is necessary to memorize, that, because of long incubation times, anti-bacterial compounds (e.g., thymol) need to be added to the incubation mixtures [19,46]. Also, dialysis or ultrafiltration can be applied as a part of the sample clean-up [49]. Finally, to exclude contamination with non-protein (and, hence, non-digested) biopolymers, hydrolysates can be ultrafiltrated with a low molecular weight (3 kDa) cut-off centrifugal filtering devices [19,46]. Additionally, to estimate the analyte losses, related to long incubations, acid hydrolysis can be set-up in parallel to the enzymatic incubation [19].

### 2.3. Analysis of Protein-Bound and Free Glycation Adducts by HPLC-ESI-MS

Although, the composition of protein hydrolysates and free glycation adducts can be addressed by GC-MS [41], typically, analysis of glycation products relies on high-performance liquid chromatography-mass spectrometry (HPLC-MS) and, most often, electrospray ionization (ESI) [76] (Table 1). Thereby, in absolute majority of cases, separation relies on one of the four most established techniques: (*i*) reversed phase (RP)-HPLC after appropriate derivatization of free amino acid adducts, also obtained in protein hydrolysates, (*ii*) ion pair (IP)-RP-HPLC, (*iii*) hydrophilic interaction liquid chromatography (HILIC), and (*iv*) chromatography on carbon columns.

Derivatization techniques are employed in LC-MS analysis of glycation products since decades. Thus, Thornalley and co-workers used aminoquinolyl-*N*-hydroxysuccinimydyl-carbamate (AQC) for detection of glycation adducts in glycated albumin with limits of detection (LODs) of several picomols [49,59] (Table 1). This compound forms derivatives, which can be detected by fluorescence (Ex/Em wavelengths of 250/395 nm) and absorption (248 nm) [112]. Essential disadvantages of this method are long analysis times and a relatively low analytical resolution, limited by the resolution of chromatographic system. Chevalier et al. applied derivatization with phenylisothiocyanate (PITC) to the analysis of in vitro glycated β-lactoglobulin (BLG) [98]. This method turned to be rather insensitive: the authors could not detect any glycated amino acids, although decrease in the contents of unglycated lysines could be confirmed. Analysis of CML, formed in vivo in plasma proteins, was successfully accomplished by RP-HPLC of acid hydrolysates treated with 9-fluorenylmethoxycarbonyl (Fmoc) chloride [119]. This method, relying on fluorescence detection (Ex/Em wavelengths of 260/310 nm) was calibrated by standard addition. Recently, it was extended to analysis of CEL and tandem mass spectrometric (MS/MS) detection [120]. Unfortunately, as acid hydrolysis was used, this protocol was not suitable for analysis of acid-labile AGEs. The same is the true for the method of Hartkopf et al. relying on the derivatization of CML with *o*-phthaldialdehyde (OPA) in acidic hydrolysates [100]. Similarly, Ehrlich and co-workers, described analysis of CML as *N^α^*-(2,4-dinitro-5-fluorophenyl)-l-valinamide (l-VDVA) derivatives in acidic collagen hydrolysates [121,122]. It is necessary to note, however, that all these methods can be easily extended to a much wider range of AGEs if adapted to enzymatic hydrolysis. One of the most sensitive methods, reported recently for quantification of AGEs, is derivatization with 2,4,6-trinitrobenzene sulfonate followed with LC-MS/MS analysis in the multiple reaction monitoring (MRM) mode. This method was successfully applied to the analysis of free glycation adducts and resulted in detection limits as low as 10 fmol [123].

It is important to note, that some AGEs can be retained on reversed phase without derivatization. Thus, Lederer and Klaiber performed such separations for 2-ammonio-6-([2-[(4-ammonio-5-oxido-5-oxopentyl)amino]-4,5-dihydro-1*H*-imidazol-5-ylidene]amino)-hexanoate (GODIC) and 2-ammonio-6-([2-[(4-ammonio-5-oxido-5-oxopentyl) amino]-4-methyl-4,5-dihydro-1*H*-imidazol-5-ylidene]amino)hexanoate (MODIC), which are relatively hydrophobic [97] (Table 1). However, in some cases, a good retention can be achieved for less hydrophobic compounds as well. For example, application of chromatographic systems, containing low amounts of methanol in mobile phase allows efficient separation of such glycation products as *N^ε^*-(2-furoylmethyl)valine and *N^ε^*-(2-furoylmethyl)lysine (furosine) on C18 reversed phase [124].

Ion pair-reversed phase chromatography (IP-RPC) is another chromatographic technique, widely used in Maillard research [103] (Table 1). In contrast to the approaches, described above, it allows analyzing of glycation adducts without derivatization. Generally, ion pair reagents, such as trifluoroacetic and heptafluorobutyric acids (TFA and HFBA, respectively) are conventionally used for purification of glycation products, providing their retention on reversed phase [125,126]. Although 0.1% TFA was successfully used as an eluent modifier for separation of argpyrimidine and pentosidine on a Hypercarb™ column [65], HFBA is a more common ion pair reagent in analysis of glycated amino acids. Thus, in the beginning of the current decade, Glomb and co-workers proposed an IP-RPC method for a high-throughput analysis of a representative pattern of at least 20 individual AGEs [19]. This method, recently extended to the detection of a wider panel of amide AGEs [127], relies on a relatively high concentrations of the ion pair reagent (0.12% *v*/*v* HFBA), which allowed a good retention of the analytes with essentially varying hydrophobicity. Interestingly, despite a high concentration of a non-volatile additive, ion suppression was moderate, and high method sensitivities (LOD typically about or below 1 pmol/mg of hydrolyzed protein) could be achieved due to combination of this approach with MS/MS analysis in a MRM mode. Thereby, quantification relied on the standard addition approach [19]. Therefore, this method combines high precision and reliability with relatively low cost requirements. During the recent decade, it’s applicability to the variety of matrices was comprehensively proved [128,129,130]. When a less variety of glycation products is supposed to be analyzed, the method can be modified appropriately. Thus, depending on the hydrophobic properties of analytes and chemistry of the reversed phase, HFBA can be supplemented to the eluents in the concentrations of 0.01 [131] or even 0.005% [45]. Although it might compromise method performance and/or minimize the spectrum of reliably detected glycation products, these conditions might reduce negative effects of ion pair reagents on mass spectrometric hardware. From the other hand, to achieve a better chromatographic behavior of hydrophilic glycation products, HFBA can be replaced by nonafluoropentanoic acid (NFPA), which has a longer hydrophobic chain in its structure and results, therefore, in a better retention of analytes and advantageous peak symmetry [132].

HILIC provides another option to quantify glycation adducts without an additional derivatization step. Thus, Yamanaka et al. applied a zwitterionic column to analysis of CML in plasma of diabetic rats [102] (Table 1). A further extension of this approach was recently suggested by Nomi and co-workers, who utilized a combination of HILIC and ion exchange separation provided by an Intrada amino acid column (Imtakt Co. Ltd., Kyoto, Japan) [133]. The authors addressed the composition of free AGEs in souses and beer, and reported quantification of seven different adducts.

Finally, HPLC on carbon columns allows quantitative retention and efficient separation of a wide selection of early and advanced glycation end products [65] (Table 1). Thus, this approach can be considered as an alternative to IP-RP-HPLC strategy, described above. The method, proposed by Thornalley et al. [65], relies on two Hypercarb™ columns, aqueous buffers and reversed phase mechanism of retention. Thereby, analytes are separated either on the first (a shorter one) column or on the series of two columns, for elution of more and less hydrophobic components, respectively [134]. The hydrophobicity of the carbon material is high enough to retain most of the glycation adducts. To ensure a sufficient retention of the most hydrophilic analytes, the hydrolysates are typically loaded in the presence of an ion pair reagent (0.1% TFA). The subsequent elution relied on acetonitrile gradients in aqueous ammonium formate buffer [135]. This design of the chromatographic experiment provides an excellent coverage of analyzed glycation products and is well-compatible with MS detection. It makes this approach advantageous in comparison to the majority of other analytical strategies. Moreover, as this protocol does not include derivatization, it is fast, reliable, free of a derivatization-related bias, and does not require high costs. Also, it integrates clean-up and separation in one procedure, that might essentially increase precision of analysis and reduce time expenses without essential contamination of mass spectrometric hardware. The method is well-compatible with tandem mass spectrometric detection in MRM mode and stable isotope dilution, which provides a high sensitivity and reproducibility [136]. It is important to note, that the Thornalley’s approach was continuously improved during the last two decades, and currently is extended over more than 20 specific adducts [76]. Moreover, it covers not only glycative modifications, but also oxidation and nitration adducts, that provides a possibility for a complex characterization of non-enzymatic protein damage in various systems of different complexity. Indeed, this method was successfully applied to diagnostic screening of glycation and oxidation markers in erythrocytes and other blood cells [65], plasma [65,137,138,139], urine [65,139,140], cerebrospinal fluid [141], synovial fluid [137,138], peritoneal dialysate [140], cultured cells [139,142], plant [139] and animal tissues [139,143,144].

### 2.4. Mass Spectrometry in Detection of Glycated Adducts

Mass spectrometric analysis of non-enzymatically modified (e.g., glycated and oxidized) amino acid adducts is typically performed on line, i.e., the column effluents are directly transferred in the ionization source of a mass spectrometer. Therefore, selection of a mass analyzer and type of experiment is critical for the success of the whole analysis. As in early works separation was mostly performed with GC, detection of glycation adducts typically relied on electron (impact) ionization quadrupole MS (EI-Q-MS) [90]. In this context, due to its favorable duty cycles, selected ion monitoring (SIM) mode turned to be advantageous in comparison to the conventional full scan (so-called, full-MS) option [93]. However, already in 1990s, liquid chromatography (LC) became the main methodological tool in analysis of glycation adducts [145]. Thereby, MS analysis relied on ion trap (IT) [121], quadrupole-time of flight (QqTOF) [124], and triple quadrupole (QqQ) [146,147] mass analyzers, operated either in a full-MS [122], or in a multiple reaction monitoring (MRM) modes [19,148].

Although analysis in a full-MS mode might lack sensitivity, it is technically unbiased, i.e., can be applied for discovery of new products. This feature makes this approach advantageous, when analysis of model glycation systems is performed, or when appearance of unknown species cannot be excluded [149]. Application of QqTOF- or FT-MS (Fourier transform MS) is, in this case, preferred, due to a high resolving power and mass accuracy of such instruments [150,151]. The modern instrumentation of this type generates the data with sub-ppm mass accuracy, which allows assignment of molecular formula with a high precision [152]. These tentatively identified products can be further characterized by their tandem mass spectrometric patterns and nuclear magnetic resonance (NMR) [116,153]. Quantitative analysis typically relies on integration of characteristic extracted ion chromatograms (XICs) at matched retention times (*t*_R_) in combination with external calibration [122].

**Figure 4:**
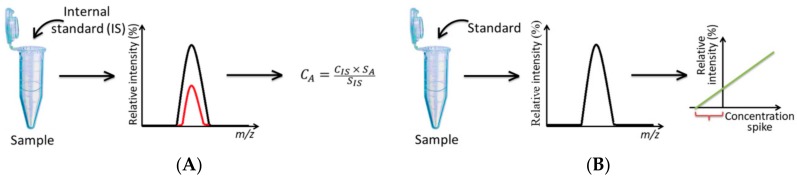
Stable isotope dilution (**A**) and standard addition (**B**) approaches for quantification of glycation adducts.

In contrast to the experiments performed in a full MS mode, MRM represents a method of highly-sensitive targeted analysis [154]. Accordingly, it can be applied to a limited number of analytes, but each of them can be detected with a high precision, accuracy and sensitivity [76]. Thereby, due to the recent improvement of the QqQ instrumentation and introduction of so-called “scheduled MRM” algorithm, which allows quantification of more than 500 analytes in one experiment [155], the power of the MRM-based targeted MS analysis dramatically increased. Moreover, introduction of the ultra-high performance liquid chromatography (UHPLC) technique in 2000s [156,157], allowed development of high-throughput quantitative LC-MS/MS-based methods, requiring in some cases only five minutes analysis times [158]. However, the most important (if not the critical) improvement of the LC-MS/MS-based quantification methodology was done by combining it with the stable isotope dilution technique [139]. This approach (Figure 4A) allows direct determination of analyte concentration (*C_A_*) in experimental samples based on the ratio of analyte (*S_A_*) and internal standard (*S_IS_*) peak areas, multiplied by the concentration of the stable isotope-labeled internal standard (*IS*) spiked to the sample *(C_IS_*) [159]:

$$C_{A}=\frac{C_{IS}\times S_{A}}{S_{IS}}$$

Thereby, due to their co-elution, both the analyte and IS are subjected to the same matrix effects, that is critical for ESI-MS [160]. Therefore, although the integrated areas of the analyte and IS can vary from injection to injection, the ratios demonstrate a good intra- and inter-day precision. During the two recent decades, this approach was comprehensively elaborated by Thornalley and co-workers [65,76,148,161], Besswinger and co-workers [146], Hashimoto et al. [123], Teerlink et al. [162] and others. Therefore the methods for synthesis of authentic and stable isotope-labeled internal standards were established [139,163,164].

It is important to note, that the standard isotope dilution method can be effectively replaced with the standard addition workflow (Figure 4B). Indeed, this technique also allows correction of matrix effects within one LC-MS/MS run. Thereby, each sample is spiked with authentic standards taken at several concentration levels (typically 3–6) to obtain a calibration curve, crossing the concentration axis at the negative range and providing a C value [143]. As was explicitly shown during the recent decade [19,101], although this technique is time consuming (as several injections per sample are required), it provides precision and sensitivity, sufficient for reliable quantification of AGEs. In the same time, it is much less expensive, and, hence, available for a higher number of laboratories.

## 3. Analysis of Glycation Adducts as a Diagnostic Tool

Even in absence of any detectable pathology, protein glycation can be observed in tissues and body fluids of living organisms at relatively low background levels [165]. However, onset of disease is often accompanied with accumulation of advanced glycation products [166]. In this context, elevated levels of specific AGE classes can serve as promising markers in diagnostics and treatment control of atherosclerosis and accompanying cardiovascular disorders, as well as DM and its complications, Alzheimer, Parkinson diseases and etc. [167]. To date, the underlying molecular mechanisms, directly affecting protein functions, are most well-characterized in the tissues of patients suffering from DM and its complications, where enhanced disease-related formation of AGEs (especially, by cross-linking) was observed [168]. Thus, accumulation of all AGE types in mammalian tissues is associated with diabetic complications, typically affecting retina, kidneys, nervous system, and blood vessels [169]. However, this phenomenon was also shown to be involved in protein aggregation related to amyloidosis, underlying, thereby, multiple neurodegenerative disorders [170]. For example, accumulation of AGEs in senile plaques located in different cortical areas of Alzheimer patient brains was described. Specifically, this phenomenon was reported for primitive plaques, coronas of classic plaques and glial cells [171,172].

In this context, development and standardization of sensitive and precise methods for qualitative and quantitative assessment of AGE patterns, accompanying pathological changes in human physiology, is required for adequate diagnostics and therapy [173]. Immunohistochemistry (IHC) is a well-established technique providing reliable and sensitive identification of AGEs in different tissues and cells [173]. For example, it allows monitoring of advanced glycation in vascularized intraocular tissues of DM patients [174]. However, although IHC technically suites well for localization of AGEs, standardized antibodies for specific AGE classes are still missing [175]. This fact essentially limits application of IHC. In contrast, enzyme-linked immunosorbent assay (ELISA) is widely used for an assessment of AGE levels in serum, plasma and other biological fluids [173]. However, although ELISA provides reliable data about CML levels in patients with complications of DM (e.g., nephropathy) [176,177,178], it has limited specificity and reproducibility [175]. Fluorescence spectroscopy is another method, often applied for characterization of advanced glycation. Thus, it was shown that the levels of skin and lens autofluorescence are much higher in DM patients in comparison to non-diabetic individuals [179,180,181]. However, this method lacks specificity, and is not applicable for detection of non-fluorescent AGEs and does not allow reliable quantitative calibration [173].

In this context, hyphenated techniques relying on GC- and LC-separation, coupled on-line to highly-sensitive fluorescence-, MS-, or MS/MS-based detection and quantification techniques are preferred for accurate and precise measurements of specific AGEs [174,182] and dicarbonyl compounds [183] in tissue and in biological fluids (Table 2). For example, using RP-HPLC in combination with fluorescence detection, Arai et al. demonstrated elevated levels of pentosidine in plasma samples obtained from patients with schizophrenia [184]. From the other hand, GS-MS was applied to quantification of CEL and CML in brains of patients suffering from Creutzfeldt-Jakob disease and Syrian hamsters affected by scrapie. In both cases, tissue contents of these AGEs were increased in comparison to healthy controls [185]. Analogously, higher contents of CML and pentosidine were confirmed in serum of DM patients by LC-MS/MS [186]. Further examples of clinically relevant quantitative analytical techniques and diagnostic markers are summarized in Table 2.

## 4. Analysis of Glycation Adducts in Foods

Accumulation of AGEs during thermal processing and prolonged storage of foods is a well-known phenomenon, comprehensively described in literature on food chemistry [189,190], and is recognized as one of the most important sources of exogenous AGEs in mammalian and human organisms [191]. Thus, numerous in vitro experiments demonstrated that consumption of AGE-rich foods might result in enhanced inflammation. Indeed, production of pro-inflammatory cytokines by cultured human endothelial cells was increased in response to food-derived AGEs [192]. The similar pro-inflammatory response was observed in vivo in the experiments with healthy human volunteers: elevated levels of vascular cell adhesion molecule 1 (VCAM-1) and C-reactive protein were associated with increased consumption of food-derived AGEs [193]. The same was the truth for type 2 DM (T2DM) patients, demonstrating a positive correlation between inflammatory markers, such as interleukin 1α (IL-1α), tumor necrosis factor α (TNF-α), monocyte chemoattractant protein-1 (MCP-1) and increased consumption of dietary AGE [194].

Analysis of various thermally processed foods revealed CML as the most abundant glycated amino acid derivative [195]. Therefore, this AGE, as well as furosine, is often used as a marker of glycation load of foods [191]. For example, based on quantification of CML by ELISA, Vlassara and co-workers proposed a database of AGE-containing foods [196,197]. However, application of GC-MS provides faster analysis, as it was done, for example, for milk and meat samples [81]. Finally, LC-MS/MS is currently the method of choice for quantitative determination of CML. Moreover, other AGE adducts can be efficiently addressed by this method as well [198]. Generally, a large variety of analyzed dietary glycation adducts can be covered by the whole pattern of analytical techniques, including not only GC- and LC-MS, but also LC coupled on-line to UV/VIS and fluorescence detection (Table 3), that in the best way can be demonstrated by analysis of heated diary products [199] and enteral formula [200]. For example, application of these techniques individually or in combination gave access to quantification of pentosidine [201], methionine sulfoxide [202], *N^ε^*-(carboxymethyl)lysine [114,161,203,204], furosine [114], *N^ε^*-(carboxyethyl)lysine [114,161], MG-H [161], and pyrraline [106].

## 5. Analysis of Glycation Adducts in Glyoxalase Research

As the majority of AGEs (at least to some extent) are formed via the autoxidative pathway, reactive dicarbonyl compounds, such as MGO and GO, are recognized as potent glycation agents [207]. Indeed, MGO reacts mainly with arginine residues to form MG-H1, CEA, argpyrimidine and THP [208], whereas GO yields CMA and Glarg [47,50]. In turn, interaction of MGO and GO with lysyl residues yields mostly CEL and CML, respectively [209]. As these adducts are the most abundant in vivo [77,210], it is obvious, that MGO and GO are the main players in the most of AGE formation pathways. Accordingly, living organisms possess an array of enzymatic and non-enzymatic defense mechanisms aimed at reducing the rates of AGE accumulation [207,211]. Among these antiglycative systems, glyoxalase system, directly involved in detoxification of MGO (and to a less extent of GO), is recognized as one of the most prominent [212]. It comprises two enzymes, namely glyoxalase-I (GLO1) and glyoxalase-II (GLO2). In the case of MGO, Glo1 catalyzes isomerization of the spontaneously formed glutathione (GSH) hemithioacetal of MGO to form a thioester (*S*-d-lactoylglutathione). In this reaction, GSH plays the role of a cofactor. In the next step, Glo2 catalyzes conversion of the thioester into d-lactate, accompanied with regeneration of GSH [213]. It was shown that Glo1 is highly conserved enzyme [214] which ubiquitously expressed in the cytosol of all cells [214,215,216,217].

Due to a high importance of the glyoxalase system, its activity is often addressed in clinical experiments, as well as in studies related to ageing, application of therapeutics and resistance of plants to environmental stress [217]. Thus, it was shown, that overexpression of Glo1 impacts in multidrug resistance, accompanying the progress of anti-cancer chemotherapy. From another hand, sensitivity of tumors to membrane-permeable Glo1 inhibitors is associated with high levels of Glo1 expression [218]. As was reported recently, drug-naive human tumors have an increased numbers of Glo1 copies, indicating existence of tumor-specific innate multidrug resistance. In this context, drugs based on Glo1 inhibitors may provide improved treatment efficiency [219]. Interestingly, alterations in activity of the both glyoxalases accompany pathogenesis of schizophrenia [184], and such neurodegenerative disorders, as Parkinson [220] and Alzheimer diseases [221]. Most likely, in general, the future studies of glyoxalase system will focus on its role in development and progression of metabolic, vascular, neurological and degenerative diseases and aging, as well as their metabolic and inflammatory regulation. Accordingly, glyoxalases themselves and related methylglyoxal-derived protein modifications may be considered as clinical biomarkers [218].

Thus, for the studies, addressing the efficiency of glyoxalase system in the context of dicarbonyl detoxification, reliable and sensitive quantitative methods are required to assess MGO-derived adducts in protein exhaustive hydrolysates. This can be achieved by two commonly used approaches: immunochemical techniques and LC-MS/MS. Thereby, immunoassays and immunostaining techniques are applied to address local variation in analyte concentrations within specific cells or tissue sections [139]. Although these methods suffer from a limited specificity of not well-defined antibodies, this problem can be to some extent solved by highly-specific monoclonal antibodies, obtained by a comprehensive screening and clone selection. In addition, quality of immunoassays can be improved by the application of synthetic (poly)peptides as blocking agents instead of commonly used milk protein [139].

However, in the context of Maillard analytics, all immunochemical methods have a common intrinsic disadvantage. Indeed, due to a high specificity of primary antibodies, quantification of only one conventional AGE class is possible within one immunochemical assay. Obviously, design of multiplexed immunoassays for dozens of AGEs will be extremely expensive and, probably, less reliable. Because of this, LC-MS/MS with stable isotope dilution as a standardization method is the technique, most often used to address this question and applicable for multi-analyte analysis [74]. Indeed, in the easiest case, protein-free adducts (i.e., the natural products of protein catabolism) can be readily quantified by LC-MS/MS in different body fluids, such as urine and plasma, after spiking with isotopically labeled internal standards [139]. For quantification of the protein glycation adducts, additional filtration and exhaustive digestion steps need to be introduced [139].

## 6. Further Perspectives

Currently, quantification of bound and free glycation adducts by LC-MS/MS in the MRM mode using the stable isotope dilution approach is a “gold standard” for analysis of early and advanced glycation products [76]. However, some considerations about the desired ways for the further development of the related analytical techniques, to our opinion, need to be addressed.

First, although the panel of conventional analytes comprises several dozens of glycation products, it is still restricted to the previously characterized structures. However, as formation of AGEs is strongly dependent from the sequence and structure moieties of corresponding proteins [108,222], their patterns might differ essentially for the samples of different composition and matrix properties. Moreover, forming AGEs can include also some structural moieties of neighboring amino acid residues [223]. Hence, the number of structural features, related to protein glycation, might be essentially higher in comparison to those conventionally considered. Therefore, untargeted profiling of glycation adducts by (U)HPLC-high resolution (HR)-MS needs to be performed to annotate the features responsive to increased carbonyl contents, and to estimate their potential for medical diagnostics and food quality assessment.

In this context, application of derivatization approaches might be advantageous for the fast and straightforward analysis of modified amino acids. Although these techniques were proposed to be less reliable in comparison to direct analysis of glycation adducts, obtained by proteolysis [76], derivatization would give an opportunity to discriminate matrix contaminations with non-amino acid compounds. For this, characteristic neutral losses, related to derivatization moiety can be diagnostic for protein-related products. Recently Milic et al. applied this strategy to characterization of carbonyl patterns, obtained by in vitro oxidation of unsaturated fatty acids [224]. The incubation mixtures were treated with 7-(diethylamino)coumarin-3-carbohydrazide, and derivatives could be unambiguously assigned by characteristic fragments at *m/z* 244 and 262. Transfer of this strategy to the analysis of amino acid glycation adducts and other derivatization agents seems to be promising, especially in combination with data analysis tools providing hierarchical clustering by characteristic class-specific fragment ions (e.g., MetFamily software, recently introduced by Balcke and co-workers [225]).

Although the reagents, conventionally used in amino acid analysis might also produce such characteristic fragments (e.g., characteristic loss of 2,4,6-trinitrobenzene moiety from 2,4,6-trinitrobenzene sulfonate derivatives of amino acids [123]), unbiased profiling of protein enzymatic digests still needs to be performed. Recently, we successfully applied different variants of unbiased MS profiling to human plasma tryptic digests, aiming identification of specific protein glycation sites associated with type 2 diabetes mellitus (T2DM) [77,80,159,226,227,228]. We believe, that analogous experiments, performed at the amino acid level, might also reveal new disease-specific markers and characteristic indicators for the loss of food quality during storage and thermal treatment. The identified markers can be, thereafter, introduced in already established LC-M/MS-based protocols, relying on stable isotope dilution [65] or standard addition [19,101] techniques. Finally, this would increase the depth of our insight in corresponding aspects of medical diagnostics and food chemistry.

Secondly, to our mind, insufficient attention is paid to the analysis of glycation adducts, originating from plant proteins. Indeed, plant-derived foods are often subjected to thermal treatment, and, generally, vegetarian diet was reported to be pro-glycative [229]. Indeed, plants demonstrate rich patterns of constitutive glycation [22]. Moreover, formation of glycation products is enhanced with plant age [230] and under stress conditions [21,231]. Remarkably, plants (especially their photosynthetically active parts) have rich patterns of potential carbonyl glycation agents, the most of which are much more reactive than glucose, dominating in mammalian blood and tissues [22]. Unfortunately, analysis of glycation adducts bound to plant proteins is a challenging task. Indeed, due to high contents of membrane organelles, the total plant proteome can be addressed only by such hard methods, as phenol extraction and acetone precipitation [232,233]. However, the isolates, obtained by these methods, cannot be quantitatively reconstituted in aqueous buffers, compatible with enzymatic digestion. According to our observations, not more than a half of the total protein is soluble in aqueous buffers. Because of this reason, currently, the proteins of plants and plant-derived foods either extracted by aqueous buffers (that is, usually, results in incomplete polypeptide degradation) [21], or the total protein sample digested by acidic hydrolysis in 6N HCl [104]. These approaches, however, cannot be considered as ideal ones: in the first case at least of the half of proteins are not covered by analysis, whereas in the second case most of the AGEs (unstable under acidic conditions) degrade during the hydrolysis. Therefore, new protocols allowing quantitative hydrolysis of the whole plant proteome are required. This would allow identification of new, plant-specific AGEs and address their physiological effects and biological role.

## Figures and Tables

**Table 1:** Overview of analytical techniques employed in analysis of free and protein-bound glycation adducts.

#	Object	Analyzed Adducts	Methodology							Ref
			Technique	Protein Isolation	Protein Hydrolysis	Derivatization (Reagents)	Separation	Detection	Standardization	
1	FFL	CML	GC-MS	-	-	Acetylation (Ac_2_O)	7%-cyanopropyl/7%-phenylmethyl coated capillary column	EI-SF, SIM	external	[41]
2	lense proteins	CML	GC-MS	cold water extraction, dialysis	acid	Acetylation (Ac_2_O)	SPB-1 capillary column (poly(dimethyl siloxane)), SE-30 capillary column (dimethylpolysiloxane) carrier helium	FID	external	[90]
3	collagen	CML, CML-OH, FL	GC-MS	CCl_4_/MeOH extraction	acid	esterification (HCL, MeOH, CH_2_Cl_2_, C_4_F_6_O_3_)	DB-5 capillary column ((5%-phenyl)-methylpolysiloxane)	EI-Q-MS, SIM	external	[91]
4	hemo-globin	CM-Ala, CM-Val, CM-Leu, CM-Ile, CM-Phe, CM-Gly, α-CML, ε-CML, bis-CML	GC-MS	-	acid	acetylation/esterification (Ac_2_O, *i*-But-OH), pentafluoro- propionic anhydride	DB-5 capillary column ((5%-phenyl)- methylpolysiloxane), DB-1701capillary column (14%-cyanopropyl- phenyl)- methylpolysiloxane) carrier helium	PICI, EI-QqQ, CAD	-	[92]
5	BSA	AGEs, poly-l-lysine	Py-GC-MS	-	pyrolysis	-	DBl capillary column (100% dimethylpolysiloxane)	EI-IT-MS	-	[95]
6	HSA	CML, CEL, MG-H, Glarg, 3-DG-H, THP, FL, pentosidine, CEL, AP, GOLD, MOLD, pyrraline	off-line HPLC-MALDI-TOF	-	enzymatic ^1^, acid	AQC	RP, analytical column NOVAPAK4 ODS (C18), NOVAPAK4 ODS (C18) Sentry guard column A: NaAc 140 mmol/L, TEA 17 mmol/L, pH 5.05, B: ACN, C: water	MALDI-TOF	internal, external	[49]
7	BSA	*N^ε^*-(1-deoxy-*d*-fructos-1-yl)-*l*-lysine	HPLC-MS	-	enzymatic ^1^	-	RP, Nucleosil 100-5 NH_2_ column (aminopropyl modified silica), A: water, B: MeOH	ESI-IT-MS	internal	[96]
8	BSA	GODIC, MODIC	HPLC-MS	-	enzymatic ^1^	-	RP, YMC- Pack Pro C 18 column, A: 10 mmol/L phosph. buffer (pH 4.0) B: MeOH, gradient	ESI-Q-MS MCA	external	[97]
9	β-lacto-globulin	Maillard reaction products	HPLC-MS	desalting and dialysis	enzymatic ^2^	-	RP, Nucleosil 300-5 C18 column, A: 0.115% aq. TFA B: 80% ACN/0.1% aq. TFA	Ex/Em: 210/330 ESI-QqQ	-	[98]
10	BSA, HSA	Pyrraline	HPLC-UV	-	alkaline	-	RP-HPLC, Vydac C18 analytical column, A: 0.1% aq. TFA, B: 50% ACN; A: 0.16% aq. HFBA, B: 0.16% aq. HFBA/50% ACN	UV, 298 nm	external	[99]
11	food samples	CML	HPLC-Fluo	-	acid	OPA	RP, Spherisorb 5 C_18_ column, A: NaAc buffer (pH 6.7, 0.05 mol/L)/4% MeOH B: MeOH	Fluo Ex/Em: 340/455	external	[100]
12	FFL	CML	HPLC-Fluo	-	-	OPA	CXC; A: 0.2 mol/L sodium citrate, pH 3.2 B: 0.2 mol/L sodium citrate, 1 mol/L NaCl pH 7.0	Fluo	-	[41]
13	lense proteins	AGEs	HPLC-Fluo	dialysis	acid, enzymatic ^3^	OPA	RP, column packed with RP-18 material A: 0.12% aq. HFBA B: 0.12% aq. HFBA/30% MeOH	Fluo Ex/Em: 340/455	external	[19]
14	lense proteins	GALA, GOLA, GOLD, CML, CMPM	HPLC-MS	dialysis	acid, enzymatic ^4^	OPA	RP, VYDAC column Knauer Eurospher 100 column RP18 A: 0.12% aq. HFBA B: 0.12% aq. HFBA/30% MeOH	ESI-Q-MS	external	[46]
15	lense proteins	AGEs	HPLC-MS/MS	dialysis	acid, enzymatic ^5^	-	RP-C18 A: 0.12% aq. HFBA B: 0.12% aq. HFBA/30% MeOH	ESI-QqQ-MS/MS, CAD, MRM	external	[19]
16	beer proteins	FL, ML, pyrraline, formyline, maltosine, MG-H1, AP	HPLC-MS/MS	dialysis	acid, enzymatic ^1^	-	RP, Zorbax 100 SB-C18 A: 10 mmol/L aq. NFPA B: 10 mmol/L aq. NFPA/ACN	ESI-QqQ-MS/MS, CAD MRM	external	[101]
17	serum	CML	LC-MS/MS	-	acid	-	HILIC (ZIC) A: 0.1% FA/ACN B: 0.1% aq. FA	ESI-QqQ-MS/MS, MRM	internal	[102]
18	food samples	α-fructosyl-amino acids	HPLC-MS	filtration	-	-	IP-RP, Kinetex core-shell C18 column A: 5 mmol/L aq, NFPA B: 5 mmol/L aq. NFPA/ACN	HESI-Orbitrap	external, internal	[103]
19	cellular and extra-cellular proteins	CML, CEL, pentosidine, GOLD, MOLD, DOLD, FL, AP, pyrraline, MG-H, 3-DG-H	HPLC-MS/MS	-	enzymatic ^1^	-	RP, Hypercarb™ columns (carbon) A: 26 mmol/L aq. AM (pH 3.8) B: 26 mmol/L aq. AM (pH 3.8)/ACN	ESI-QqQ-MS/MS CAD MRM	internal	[65]

^1^ Pepsin, pronase E, aminopeptidase, prolidase; ^2^ trypsin; ^3^ pronase E, aminopeptidase; ^4^ carboxypeptidase Y; ^5^ proteinase K, carboxypeptidase Y, peptidase, Pronase E, aminopeptidase; %, %(*v*/*v*); 3-DG-H, 3-deoxyglucosonederived hydroimidazolone; ACN, acetonitrile; AGEs, advanced glycation end products; AM, ammonium formate; AP, argpyrimidine; aq., aqueous; AQC, 6-aminoquinolyl-*N*-hydroxysuccinimidyl-carbamate; BSA, bovine serum albumin; CAD, collision-activated dissociation; CEL, *N^ε^*-(carboxyethyl)lysine; CXC, cation exchange chromatography; CM-Ala, *N*-(carboxymethyl)alanine; CM-Gly, *N*-(carboxymethyl)glycine; CM-Ile, *N*-(carboxymethyl)isoleucine; CML, *N′-*(carboxymethyl)lysine; CM-Leu, *N*-(carboxymethyl)leucine; CML-OH, *N^ε^*-(carboxymethyl)hydroxylysine; CM-Phe, *N*-(carboxymethyl)phenylalanine; CMPM, [(3-hydroxy-5-hydroxymethyl-2-methyl-pyridin-4-ylmethyl)amino]acetic acid; CM-Val, *N*-(carboxymethyl)valine; DOLD, 3-deoxyglucosone-derived lysine dimer; EI, electron (impact) ionization; ESI, electrospray ionization; Ex/Em, excitation/emission wavelengths; FA, formic acid; FID, flame ionization; FFL, Nu-formyl-*N′*-fructose-lysine; FL, fructose-lysine; GALA, *N*^6^-(glycoloyl)lysine; GC-MS, gas chromatography–mass spectrometry; Glarg, glyoxal-derived hydroimidazolone; GODIC, 2-ammonio-6-([2-[(4-ammonio-5-oxido-5-oxopentyl)amino]-4,5-dihydro-1*H*-imidazol-5-ylidene]amino)-hexanoate; GOLA, *N^ε^*-[2-[(5-amino-5-carboxypentyl)amino]-2-oxoethyl]lysine; GOLD, glyoxal-derived lysine dimer; HESI, heated electrospray ionization; HFBA, heptafluorobutyric acid; HILIC, hydrophilic interaction liquid chromatography; HPLC, high-performance liquid chromatography; HSA, human serum albumin; *i*-But-OH, isobutanal; IP, ion-pairing; IT, ion trap; LC, liquid chromatography; MALDI, matrix assisted laser desorption/ionization MCA—multichannel acquisition; MeOH, methanol; MG-H, methylglyoxal-derived hydroimidazolone; ML, maltulosyllysine; MODIC, 2-ammonio-6-([2-[(4-ammonio-5-oxido-5-oxopentyl) amino]-4-methyl-4,5-dihydro-1*H*-imidazol-5-ylidene]amino)hexanoate; MOLD, methylglyoxal-derived lysine dimer; MRM, multiple reaction monitoring; MS, mass spectrometry; MS/MS, tandem mass-spectrometry; NFPA, nonafluoropentanoic acid; ODS, octadecyl silica; OPA, o-phthaldialdehyde; PICI, positive ion chemical ionization; PITC, phenylisothiocyanate; Py-GC-MS, pyrolysis GC-MS; QMS, quadrupole mass analyzer; QqQ, triple quadrupole; RP, reversed phase; SF, sector field; SIM, selective ion monitoring; TEA, trimethylamine; TFA, trifluoroacetic acid; THP, *N^δ^*-(4-carboxy-4,6-dimethyl-5,6-dihydroxy-1,4,5,6-tetrahydropyrimidin-2-yl)-ornithine; UV, ultra-violet detection; *v*/*v*, ratio by volume; #, number.

**Table 2:** Application of glycation adduct analysis in medical diagnostics.

#	Disease	Object	Analyzed Adducts	Main Results	Methodology							Ref.
					Technique	Protein Isolation	Protein Hydrolysis	Derivatization	Separation	Detection	Standardization	
1	T1DM	serum	CML, pentosidine	increase of AGE levels	HPLC-MS/MS	serum treatment	acid	-	Kinetex HILIC/PFP (CML/pentosidine) A: 5 mmol/L aq. AM B: 100% ACN	ESI-QqQ-MS/MS CAD MRM	internal	[186]
2	DM	rabbit blastocyst cavity fluid	CML	increase of CML levels	HPLC-MS/MS	analysis of free adducts	-	-	RP, C18 A: 0.12% aq. HFBA B: 0.12% aq. HFBA/30% MeOH	ESI-QqQ-MS/MS CAD MRM	internal	[130]
3	DM	rat plasma protein	CML, CEL, Glarg, MG-H1	increase of AGE levels	HPLC-MS/MS	Ultrafiltra-tion (12 kDa cut-off)	enzymatic ^1^	-	RP, carbon Hypercarb^™^ A: 26 mmol/L AM, pH 3.8, B: ACN	ESI-QqQ-MS/MS CAD MRM	internal	[65]
4	diabetic nephro-pathy	blood from normoalbuminuric subjects (NHDNS)	CML, CEL, MG-HI	increase of AGE levels	HPLC-MS/MS	filtration (10 KDa cutoff)	-	-	RP, C18 Synergy 80 A A: 0.29% aq. HFBA, B: 0.29% aq. HFBA/MeOH	ESI-QqQ-MS/MS CAD MRM	internal	[187]
5	fibrosis	human aged lens capsules	CML, NFL, CMA, NAL, CEA, MG-H1, Pyrraline Glucosepane,MODIC	AGEs in the lens capsule promote fibrosis of lens epithelial cells	HPLC-MS/MS		enzymatic ^2^	-	RP, C18 A: 0.12% aq. HFBA B: 0.12% aq. HFBA/30% MeOH	ESI-QqQ-MS/MS CAD MRM	internal	[128]
6	cataract	lense proteins	CML, MG-HI	increase of CML levels	HPLC-MS/MS	phosphate-buffered Saline/EDTA dialysis	acid, enzymatic ^3^	OPA	RP, C18, A: 0.12% aq. HFBA B: 0.12% aq. HFBA/30% MeOH	ESI-QqQ-MS/MS, CAD MRM	external	[19]
7	prion disease	Creutzfeldt- Jakob/brain scrapie/Syrian hamsters	CML, CEL	elevated AGE levels in plaques	GS-MS	CHCl_3_- CH_3_OH extraction	acid	esterify-cation (HCL, MeOH, CH_2_Cl_2_, C_4_F_6_O_3_)	HP-5MS column	EI-Q-MS	internal	[185]
8	schizophrenia	plasma/schizophrenia	pentosidine	elevated level of AGEs	IP-RP- HPLC-Fluo	-	acid	-	RP, C18 A: 0.1% aq.HFBA B: 0.1% aq.HFBA/ACN	Fluo Ex/Em: 335/385 nm	external	[184]
9	schizophrenia	plasma/schizophrenia	pentosidine	elevated level of AGEs	IP-RP- HPLC-Fluo	-	acid	-	RP, C18 A: 0.1% aq. HFBA B: 0.1% aq. HFBA/ACN	Fluo Ex/Em 335/385 nm	external	[188]

^1^ Pepsin, pronase E, aminopeptidase, prolidase; ^2^ collagenase, pronase E; ^3^ pronase E, leucine aminopeptidase, carboxypeptidase Y; %, %(*v*/*v*); ACN, acetonitrile; AGE, advanced glycation end products; AM, ammonium formate; CAD, collision-activated dissociation; CEA, *N*^6^-(carboxyethyl)arginine; CEL, *N^ε^*-(carboxyethyl)lysine; CMA, *N*^6^-(carboxymethyl)arginine; CML, *N^ε^*-(carboxymethyl)lysine; DM, Diabetes mellitus; DOLD, 3-deoxyglucosone-derived lysine dimer, 1,3-di(*N^ε^*-lysino)-4-(2,3,4-trihydroxybutyl)-imidazolium salt; EI, electron ionization; Ex/Em, excitation/emission wavelengths; ESI, electrospray ionization; FL, *N^ε^*-(fructosyl)lysine; Glarg, *N^δ^*-(5-hydro-4-imidazolon-2-yl)ornithine; GOLD, glyoxal-derived lysine dimer, 1,3-di(*N^ε^*-lysino)imidazolium salt; Fluo, fluorescent detection; GS-MS, gas chromatography–mass spectrometry; HFBA, heptafluorobutyric acid; HILIC, hydrophilic interaction liquid chromatography; HPLC, high-performance liquid chromatography; LC, liquid chromatography; MALDI-TOF, matrix assisted laser desorption/ionization time-of-flight; MeOH, methanol; MG-H1, (*N^δ^*-(5-hydro-5-methyl-4-imidazolon-2-yl)-ornithine); MODIC, 2-ammonio-6-({2-[(4-ammonio-5-oxido-5-oxopentyl)amino]-4-methyl-4,5-dihydro-1*H*-imidazol-5-ylidene}amino)hexanoate; MOLD, methylglyoxal-derived lysine dimer; MRM, multiple reaction monitoring; MS, mass spectrometry; MS/MS, tandem mass spectrometry; NAL, *N*^6^-acetyllysine; NFL, *N*^6^-(formyl)lysine; NHDNS, Natural History of Diabetic Nephropathy Study; OPA, *o*-phthaldialdehyde; PFP, pentafluorophenyl; QMS, quadrupole mass analyzer; QqQ, triple quadrupole; RP, reversed phase; T1DM, Diabetes mellitus type 1; TFAME, trifluoroacetyl methyl ester; #, number.

**Table 3:** Application of glycation adduct analysis in food research.

#	Type of Food	Analyzed Adducts	Methodology							Ref.
			Technique	Protein Isolation	Protein Hydrolysis	Derivatization	Separation	Detection	Standardization	
1	milk products	CML	RP-HPLC -Fluo	Direct hydrolysis after reduction with 1 mol/L NaBH_4_	acid	OPA	RP, C18 SpheriChROM RP-18 ODS, A: sodium acetate buffer (pH 6.50, 0.048 mol/L)/4% MeOH B: MeOH	Fluo Ex/Em: 340/455 nm	standard addition external	[205]
2	milk products	CML, CEL, MG-H, FL, Argpyrimidine, 3DG-H, DOLD, Glarg, GOLD, MOLD	HPLC- MS/MS	Ultrafiltration (12 kDa cutoff) delipidation	enzymatic ^1^	-	Hypercarb™ A: 26 mmol/L AM (pH 3.8) B: ACN	ESI-QqQ- MS/MS CAD MRM	internal	[161]
3	milk products	CML	GS-MS	extraction: C_2_H_6_O-CH_2_Cl_2_	acid	MeOH/TFAA	DB5-MS capillary column carrier helium	EI-IT-MS	internal, external, isotope dilution	[81]
4	milk products	CML, furosine, CEL	HPLC–MS/MS	Hydrolyzed without protein isolation	acid	-	RP, C18 core shell Kinetex A: 5 mmol/L PFPA B: 5 mmol/L aq. PFBA/ACN	ESI-QqQ- MS/MS CAD MRM	internal	[114]
5	milk products	CML	UHPLC–MS/MS	precipitation: TCA/extraction: CHCl_3_-MeOH	acid	-	RP, C18 Acquity UPLC™ BEC C_18_ column A: 0.13% aq. NFPA or 0.1% aq. TFA B: ACN	ESI-QqQ-MS/MS MRM	internal	[201]
6	bakery products	CML, furosine, CEL	HPLC– MS/MS	-	acid	-	RP, C18 core shell Kinetex A: 5 mmol/L aq. PFPA B: 5 mmol/L aq. PFBA/ACN	ESI-QqQ- MS/MS CAD MRM	internal	[114]
7	bakery products	CML	GS-MS	extraction: CHCl_3_-MeOH	acid	MeOH/TFAA	DB5-MS capillary carrier helium	EI-IT-MS	internal, external, isotope dilution	[81]
8	bakery products	CML	HPLC–MS/MS	precipitation: TCA/extraction: CHCl_3_- MeOH	acid	-	RP, C18 Acquity BEH C_18_ column A: 0.13% aq NFPA or 0.1% aq. TFA B: ACN	ESI-QqQ- MS/MS CAD MRM	internal	[201]
9	meat	CML	GS-MS	extraction: CHCl_3_-MeOH	acid	esterification by MeOH/acylation by TFAA	DB5-MS capillary carrier helium	EI-IT-MS	internal, external, isotope dilution	[81]
10	meat	CML	HPLC–MS/MS	precipitation: TCA/extraction: CHCl_3_-MeOH	acid	-	RP, C18 Acquity BEH C_18_ col. A: 0.13% aq. NFPA or aq. 0.1% TFA B: ACN	ESI-QqQ- MS/MS CAD MRM	internal	[201]
11	fish	CML	GS-MS	extraction: CHCl_3_-MeOH	acid	esterification by MeOH/acylation by TFAA	DB5-MS capillary carrier helium	EI-IT-MS	internal, external, isotope dilution	[81]
12	coffee	melanoidins	Off line LC-MALDI-TOF-MS	Hot water extraction delipidation	-	-	GFC Sephadex G-25	MALDI- TOF-MS	external	[206]

^1^ Pronase E, aminopeptidase, prolidase; %, % (*v*/*v*); ACN, acetonitrile; 3DG-H, *N^δ^*-(5-hydro-5-(2,3,4-trihydroxybutyl)-4-imidazolon-2-yl) ornithine and related structural isomers; CEL, *N^ε^*-(carboxyethyl)lysine; CML, *N^ε^*-(carboxymethyl)lysine; DOLD, 3-deoxyglucosone-derived lysine dimer, 1,3-di(*N^ε^*-lysino)-4-(2,3,4-trihydroxybutyl)-imidazolium salt; EI, electron ionization; ESI, electrospray ionization; FL, fructosyl-lysine; Fluo, fluorescent detection; IT, ion trap; GFC, gel-filtration chromatography; Glarg, *N^δ^*-(5-hydro-4-imidazolon-2-yl)ornithine; GOLD, glyoxal-derived lysine dimer, 1,3-di(*N^ε^*-lysino)imidazolium salt; GS-MS, gas chromatography–mass spectrometry; HPLC, high-performance liquid chromatography; LC, liquid chromatography; MALDI-TOF, matrix assisted laser desorption/ionization time-of-flight; MeOH, methanol; MG-H1, (*N^δ^*-(5-hydro-5-methyl-4-imidazolon-2-yl)-ornithine); MOLD, methylglyoxal-derived lysine dimer; MRM, multiple reaction monitoring; MS, mass spectrometry; MS/MS, tandem mass spectrometry; NFPA, nonafluoropentanoic acid; OPA, *o*-phthaldialdehyde; PFPA, perfluoropentanoic acid; RP, reversed phase; QqQ, triple quadrupole; TCA, trichloroacetic acid; TFA, trifluoroacetic acid; TFAA, trifluoroacetic acid anhydride; UHPLC, ultra-high-performance liquid chromatography.

## Abbreviations

3-DG	3-deoxyglucosone
3-DG-H	3-deoxyglucosone-derived hydroimidazolone
3-DP	3-deoxypentosone
ACN	acetonitrile
AGEs	advanced glycation end products
AM	ammonium formate
AP	argpyrimidine
aq.	aqueous
AQC	6-aminoquinolyl-*N*-hydroxysuccinimidyl-carbamate
BAC	boronic acid affinity chromatography
BLG	β-lactoglobulin
BSA	bovine serum albumin
CAD	collision-activated dissociation
CEA	*N^δ^*-(carboxyethyl)arginine
CEL	*N^ε^*-(carboxyethyl)lysine
CMA	*N^δ^*-(carboxymethyl)arginine
CM-Ala	*N^α^*-(carboxymethyl)alanine
CM-Gly	*N^α^*-(carboxymethyl)glycine
CM-Ile	*N^α^*-(carboxymethyl)isoleucine
CML	*N^ε^*-(carboxymethyl)lysine
CM-Leu	*N^α^*-(carboxymethyl)leucine
CML-OH	*N^ε^*-(carboxymethyl)hydroxylysine
CM-Phe	*N^α^*-(carboxymethyl)phenylalanine
CMPM	[(3-hydroxy-5-hydroxymethyl-2-methyl-pyridin-4-ylmethyl)amino]acetic acid
CM-Val	*N^α^*-(carboxymethyl)valine
CXC	cation exchange chromatography
DM	diabetes mellitus
DOLD	3-deoxyglucosone-derived lysine dimer
EI	electron (impact) ionization
ELBIA	enzyme-linked boronate-immunoassay
ELISA	enzyme-linked immunosorbent assay
ESI	electrospray ionization
Ex/Em	excitation/emission wavelengths
FA	formic acid
FFL	*N^α^*-formyl-*N^ε^*-(fructosyl)lysine
FID	flame ionization detector
FIP-U	Fédération Internationale Pharmaceutique unit
FL	*N^ε^*-(fructosyl)lysine
Fmoc	9-fluorenylmethoxycarbonyl
FT-MS	Fourier transform MS
GALA	*N^ε^*-(glycoloyl)lysine
GC-MS	gas chromatography–mass spectrometry
GFC	gel-filtration chromatography
GLAP	glyceraldehyde-derived pyridinium compound
Glarg	1-(4-amino-4-carboxybutyl)-2-imino-5-oxo-imidazolidine, glyoxal-derived hydroimidazolone
GLO1	glyoxalase-I
GLO2	glyoxalase-II
GO	glyoxal
GODIC	2-ammonio-6-([2-[(4-ammonio-5-oxido-5-oxopentyl)amino]-4,5-dihydro-1H-imidazol-5-ylidene]amino)-hexanoate
GOLA	*N^ε^*-[2-[(5-amino-5-carboxypentyl)amino]-2-oxoethyl]lysine
GOLD	glyoxal-derived lysine dimer
GSH	glutathione
HbA_1C_	glycated hemoglobin
HESI	heated electrospray ionization
HFBA	heptafluorobutyric acid
HILIC	hydrophilic interaction liquid chromatography
HPLC	high-performance liquid chromatography
HR-MS	high resolution MS
HSA	human serum albumin
*i*-but-OH	isobutanol
IHC	immunohistochemistry
IL-1α	interleukin 1α
IP-RPC	ion pair-reversed phase chromatography
IS	internal standard
IT	ion trap
LC	liquid chromatography
LOD	limit of detection
L-VDVA	*N^α^*-(2,4-dinitro-5-fluorophenyl)-l-valinamide
MALDI	matrix assisted laser desorption/ionization
MCA	multichannel acquisition
MCP-1	monocyte chemoattractant protein-1
MeOH	methanol
MG-H	methylglyoxal-derived hydroimidazolone
MG-H1	*N^δ^*-(5-methyl-4-oxo-5-hydroimidazo-linone-2-yl)-l-ornithine
MG-H2	2-amino-5-(2-amino-5-hydro-5-methyl-4-imidazolon-1-yl)pentanoic acid
MG-H3	2-amino-5-(2-amino-4-hydro-4-methyl-5-imidazolon-1-yl)pentanoic acid
MGO	methylglyoxal
ML	*N^ε^*-(maltosyl)lysine
MODIC	2-ammonio-6-([2-[(4-ammonio-5-oxido-5-oxopentyl)amino]-4-methyl-4,5-dihydro-1*H*-imidazol-5-ylidene]amino)hexanoate
MOLD	methylglyoxal-derived lysine dimer
MRM	multiple reaction monitoring
MS	mass spectrometry
MS/MS	tandem mass-spectrometry
NAL	*N^ε^*-(acetyl)lysine
NFL	*N^ε^*-(formyl)lysine
NFPA	nonafluoropentanoic acid
NHDNS	Natural History of Diabetic Nephropathy Study
NMR	nuclear magnetic resonance
ODS	octadecyl silica
OPA	*o*-phthaldialdehyde
PFP	pentafluorophenyl
PFPA	perfluoropentanoic acid
PICI	positive ion chemical ionization
PITC	phenylisothiocyanate
PU	papain units
Py-GC-MS	pyrolysis GC-MS
Q	quadrupole mass analyzer
QqQ	triple quadrupole
QqTOF	quadrupole-time of flight
RAGEs	receptors to advanced glycation end products
RP	reversed phase
SF	sector field
SIM	selected ion monitoring
T1DM	type 1 diabetes mellitus
T2DM	type 2 diabetes mellitus
TCA	trichloroacetic acid
TEA	trimethylamine
TFA	trifluoroacetic acid
TFAME	trifluoroacetyl methyl ester
THP	*N^δ^*-(4-carboxy-4,6-dimethyl-5,6-dihydroxy-1,4,5,6-tetrahydropyrimidin-2-yl)-ornithine
TNF-α	tumor necrosis factor α
UHPLC	ultra-high performance liquid chromatography
UV	ultra-violet
VCAM-1	vascular cell adhesion molecule 1
VIS	visual light
*v*/*v*	volume/volume
XIC	extracted ion chromatogram
